# Autonomic activity and cardiovascular system risk assessment in pediatric patients with hemolytic uremic syndrome

**DOI:** 10.1007/s00431-024-05420-x

**Published:** 2024-01-19

**Authors:** Derya Duman, Serra Sürmeli Döven, Derya Karpuz, Esra Danacı Vatansever, Bahar Taşdelen, Ali Delibaş

**Affiliations:** 1 Faculty of Medicine, Department of Pediatric Cardiology, Mersin University, 34. Cadde, Ciftlikkoy Kampusu, 33343 Mersin, Turkey; 2 Faculty of Medicine, Department of Pediatric Nephrology, Mersin University, 34 Cadde, Ciftlikkoy Kampusu 33343, Mersin, Turkey; 3 Faculty of Medicine, Department of Biostatistics and Medical Informatics, Mersin University, 33343 Mersin, Turkey

**Keywords:** Arrhythmia, Autonomic nervous system, Heart rate variability, Hemolytic uremic syndrome

## Abstract

In pediatric patients with hemolytic uremic syndrome (HUS), cardiac involvement and autonomic nervous system function can be evaluated by a non-invasive method called heart rate variability (HRV). This study aims to evaluate heart rate variability and electrocardiography findings in patients with HUS by comparing a healthy group. Patients who are diagnosed with HUS at a university hospital from December 2020 to June 2022 are screened by electrocardiography (ECG), echocardiography, and 24-h Holter ECG. A healthy control group, compatible in age and gender with the patient group, was selected from healthy subjects. HRV parameters, laboratory values, and ECG findings were analyzed and compared with the healthy group and each other. There were 25 patients with HUS and 51 participants in the healthy control group. Statistically significant differences were found in some HRV parameters: standard deviation of normal to normal intervals, the mean of the 5-min RR interval standard deviations, the standard deviation of 5-min RR interval means, the triangular interpolation of normal to normal interval, and very-low-frequency power. HUS patients had impaired and declined HRV values compared to the healthy group. There was a significant decrease in the PR distance, while a significant increase in the corrected QT and QT dispersion values was detected in the electrocardiographic findings of the patient group. HRV values impaired as renal failure parameters increased.

*  Conclusion:* Patients with HUS may have autonomic nervous system dysfunction. HRV measurement is a non-invasive method that can evaluate this. It can be thought that there may be an increased risk of cardiovascular events and arrhythmias in some patients with HUS. ECG should be also considered to detect arrhythmia.

**Table:** 

**What is Known:** *• Hemolytic uremic syndrome (HUS) primarily effects the hematologic parameters and kidney.* *• Secondary cardiomyopathy with hypertension and renal failure could be observed in these patients.* *• Rhythm problems are not expected primarily in these patients.* *• There is very limited data in evaluating autonomic function and arrhythmia risk for these patients.*	
**What is New:** *• Patients with HUS may have autonomic nervous system dysfunction.* *• HRV measurement is a non-invasive method that can evaluate this.* *• Cardiovascular events and arrhythmias due to the deterioration of the balance between the sympathetic and parasympathetic systems could manifest in patients with HUS.* *• An ECG and screening patients for cardiac events, and monitoring them closely should be considered.*

**What is Known:**

*• Hemolytic uremic syndrome (HUS) primarily effects the hematologic parameters and kidney.*

*• Secondary cardiomyopathy with hypertension and renal failure could be observed in these patients.*

*• Rhythm problems are not expected primarily in these patients.*

*• There is very limited data in evaluating autonomic function and arrhythmia risk for these patients.*

**What is New:**

*• Patients with HUS may have autonomic nervous system dysfunction.*

*• HRV measurement is a non-invasive method that can evaluate this.*

*• Cardiovascular events and arrhythmias due to the deterioration of the balance between the sympathetic and parasympathetic systems could manifest in patients with HUS.*

*• An ECG and screening patients for cardiac events, and monitoring them closely should be considered.*

## Introduction

Hemolytic uremic syndrome (HUS) is described as the concurrent existence of microangiopathic hemolytic anemia, thrombocytopenia, and acute kidney injury [1]. Kidney injury could lead to several manifestations including mortality. HUS can be classified into two types, depending on the presence of a diarrheal prodrome. HUS without diarrhea is called atypical HUS, and the etiology usually includes an underlying predisposing genetic factor [2].

As with most systemic diseases, cardiac involvement can also be observed here. In particular, secondary cardiomyopathy with hypertension and renal failure and also the involvement of the heart muscle due to microvascular damage are more pronounced in these patients with the atypical form [3]. Although rhythm problems are not expected primarily in these patients, arrhythmias such as ventricular tachycardia may be present in some cases [4].

The autonomic nervous system consists of sympathetic and parasympathetic components. With a disorder in the autonomic nervous system, other body systems may also be affected, resulting in various undesirable effects. Autonomic nervous system activity can be calculated using heart rate variability (HRV), a non-invasive method [5]. In patients with impaired HRV, the prognosis of the disease may be worse due to the risk of arrhythmia as well as other systems being affected. The central nervous system may also be affected by autonomic neuropathy [6].

Electrocardiographic (ECG) findings could also have a such important impact on the prognosis of the disease; there are very limited data in evaluating the ECG of these patients.

To our current knowledge, there are no studies evaluating the functions of the autonomic nervous system in pediatric patients with HUS. In this study, it was aimed to evaluate autonomic nervous system function by performing HRV analyses after the acute period in pediatric patients diagnosed with HUS. Electrocardiographic findings were also determined with HRV evaluation.

## Methods

Patients diagnosed with HUS, at a university hospital, from December 2020 to June 2022, were included in this study. The control group, who were compatible in age and gender with the patient groups, were selected from healthy subjects who were admitted to the hospital for a murmur, chest pain, or palpitation. The local ethics committee approved the protocol. The Helsinki declaration was taken into consideration. The study was conducted in accordance with the Declaration of Helsinki. Written informed consent was acquired from the parents of all of the participants.

All of the patients in the study had appropriate diagnostic features of HUS, including laboratory studies, and genetic study findings in some patients [1]. Inclusion criteria were having been diagnosed and followed up in this single-center clinic. None of HUS patients received any intra-venous treatment during 24-h Holter ECG. They were analyzed after the acute period of the disease, late after treatment before discharge from hospital.

The inclusion criteria for the control group were the absence of other cardiologic diseases including congenital structural heart diseases and arrhythmia syndromes.

The exclusion criteria for patients and the control group were that they had other systemic diseases.

Electrocardiography (standard 12-lead ECG) was carried out for the participants at a paper velocity of 25 mm/s under close circumstances. A Nihon Kohden ECG 1250 Cardio fax S (2009, Tokyo, Japan) tool was utilized at standard velocity and amplitude, with 24-h Holter ECG analysis (Century Holter model 3000 system). Transthoracic echocardiography was achieved with a Vivid E9 Pro Ultrasound System (GE Medical Systems, Canada) using 3- and 6-MHz transducers as 2D, M-mode and colored Doppler, conventional continuous-wave (CW), and pulse-wave (PW) Doppler imagining ways. Two experienced pediatric cardiologists performed all of the examinations.

The electrocardiography visions had a 600-dpi imaging quality and assessments were made on a computer by a practiced pediatric cardiologist. ECG parameters, comprising heart rate; P wave; PR interval; QRS axis, duration, and morphology; T-wave voltages and polarity; ST-segment changes; and QT interval (corrected by heart rate according to the Bazzett formula QTc = QT/√RR [s], where RR is stated in the foregoing RR interval), were manually analyzed and measured. The tangent method is used in QT measurement [7]. Furthermore, the heart rate, Tpeak–end (Tp-e), Tp-e dispersion, and Tp-e/QT ratio were evaluated. The TpTe was measured via the tangent method in precordial derivations [8]. The QT dispersion (QTd) is the difference between QT max and QT min on the surface ECG that reflects the time from the start of depolarization to the end of repolarization. Ventricular refractoriness could be measured by this way.

Recordings taken with a vx3 + model Century Holter model 3000 system solid-state recorder were evaluated via computer using the same software system. None of the patients received inotropes or other cardiovascular medications like beta-blockers or other antiarrhythmic therapy during the 24-h Holter ECG monitoring.

### Heart rate variability analysis

HRV is a basic, non-invasive, equitable, and confirmed measuring mechanism for the evaluation of autonomic nervous system function [9, 10]. HRV is acquired from intervals between normal sinus heartbeats (NNs) and can be quantified using a variety of methods [5, 9, 11, 12], primarily comprising time domain measures, frequency domain measures, and heart rate turbulence. Time domain and frequency domain approaches are based on the calculation of differences in connected RR intervals on 24-h Holter ECG enrollments.

#### Time domain study

Evaluations were managed on the alteration of the heart rate during a standard time interval based on RR distances between two consecutive sinus beats. These values included the standard deviation of normal to normal intervals (SDNN), the standard deviation of 5-min RR interval means (SDANN), the mean of the 5-min RR interval standard deviations (SDNNi), root mean square of successive RR interval differences (rMSSD), and the percentage of the beats with a consecutive RR interval difference of more than 50 ms (pNN50). The HRV triangular index (HRV TI) was acquired as the total number of all of the RR intervals divided by the height of the histogram and TINN as baseline width of the minimum square difference triangular interpolation of the highest peak of the histogram.

#### Frequency domain study

This was evaluated using cyclic marks, with an average of 500 sequential RR intervals divided into different bands of frequency answer. Total power (the area under the spectral curve from 0.01 to 1.0 Hz, TP), very-low-frequency power (the area under the spectral curve from 0.0033 to 0.04 Hz, VLF), low-frequency (the area under the spectral curve from 0.04 to 0.15 Hz, LF), and high-frequency band power (the area under the spectral curve from 0.15 to 0.40 Hz, HF) were inspected and the LF/HF ratio was measured.

Frequency domain parameters include low-frequency (LF) and high-frequency (HF) bands in spectral analysis. LF bands are connected to the sympathetic nervous system, including the parasympathetic component. VLF bands are bonded to parasympathetic deactivation [13]. The HF reflects parasympathetic action, whereas LF shows both sympathetic and parasympathetic action, and SDNN, rMSSD, and pNN50 define the parasympathetic action [13]. The LF is the sole parameter used to calculate the activity of sympathetic activity. The LF/HF index is between 0.15 and 0.4. It is related to the modulation of the efficacy of the gas exchange, respiratory sinus arrhythmia, parasympathetic nervous system activity, and innervation of the vagus nerve.

To evaluate HRV parameters in this study, a healthy control group was determined and compared with the patient group. A decrease in HRV compared to the healthy patients was considered significant evidence for corruption in HRV. Premature ventricular and supraventricular beats were eliminated in the HRV analysis.

### Statistical analysis

Categorical data were expressed as numbers and percentages. Normal distribution control of continuous data was done with the Shapiro–Wilk test. Continuous data not suitable for normal distribution according to mean and standard deviation data were expressed as the median and quartiles. Group comparisons were made with the independent two-group *t* test or Mann–Whitney *U* test. Then, multiple logistic regression analysis was done to evaluate the multivariable significance of the univariate significant variables.

In addition, the relationships between the HRV parameters and laboratory and echocardiographic measurements were evaluated with Spearman correlation coefficients. Statistically significant correlations greater than 0.40 were interpreted. Diagnostic performances of HRV and ECG parameters were evaluated using receiver operating curve analysis. The sensitivity and specificity values for the obtained cutoff values were summarized. Statistical significance was accepted as *P* < 0.05. Statistical analyses were performed with the STATISTICA 13.0 package program. Power analysis was made to define the patient and control group numbers.

## Results

There were 25 patients diagnosed as HUS and 51 patients in the control group. The mean age was 77.120 ± 59.176 (11–132) months in the HUS group and 95.216 ± 56.447 (10–156) months in the control group. Twelve patients were diagnosed with atypical HUS. Cardiac biomarkers were in the normal range. In the acute period, the median hemoglobin level was 7.2 g/dL (1–12 g/dL), the median platelet level was 76.2 × 10^3^/μL (8–140 × 10^3^/μL), median white blood level count was 16.8 × 10^3^/μL (4.2–56 × 10^3^/μL), median C-reactive protein (CRP) level was 40.5 mg/L (15–203 mg/L: upper limit was 5 mg/L), the creatinine median level was 2.4 mg/dL (0.6–13.4 mg/dL), median urea level was 146 m/dL (42.5–600 mg/dL), median sodium level was 133 mEq/L (119–138 mEq/L), median potassium level was 4.56 mEq/L (2.86–6.79 mEq/L), median calcium level was 8.2 mg/dL (6.92–10 mg/dL), median LDH level was 2100 U/L (741–3638 U/L), and median haptoglobin level was 0.02 g/L (0.00–0.25 g/L) in patients with HUS. All patients had dysmorphic erythrocytes and erythrocyte casts in their urine, along with an appearance consistent with glomerulonephritis and proteinuria.

After the acute period, the median creatinine level was 0.57 mg/dL (0.15–3.62 mg/dL), and the control urea median level was 32.4 mg/dL (6.4–87.6 mg/dL). The median TSH level was 2.5 μIU/mL (0.16–5 μIU/mL, normal ranges were 0.73–8.35 μIU/mL), and median free T4 (fT4) was 11.7 pmol/L (8.3–16 pmol/L, normal ranges were 8–22 pmol/L). The TSH, fT4, CRP, and electrolyte levels were normal when the HRV analysis was analyzed. Systolic and diastolic blood pressures were higher in the HUS patients than in the control group. The baseline characteristics of all of the participants are summarized in Table 1.

**Table 1:** Demographic features and physical examination results of the groups

	HUS Mean ± SD	Control group Mean ± SD	***P* value**
Age (months)	77,120 ± 59,176	95,216 ± 56,447	**0.114**
Gender			
M, *n* (%)	16 (64%)	30 (58.8%)	
F, *n* (%)	9 (36%)	21 (41.1%)	
Systolic blood pressure mmhg	116,840 ± 16,454	104,902 ± 10,513	**0.001**
Diastolic blood pressure mmhg	75,600 ± 13,191	67,098 ± 9233	**0.011**
Height (centimeter)	116,920 ± 31,123	122,392 ± 28,555	**0.514**
Weight (kilogram)	27,336 ± 19,662	28,618 ± 17,592	**0.599**

*N* number, *HUS* hemolytic uremic syndrome

The echocardiographic evaluations of all of the control participants were normal. The left ventricular systolic function was slightly decreased in 5 patients with HUS (45–50%) and there was a left ventricular trabeculation increase with left ventricular hypertrophy in all of the patients with HUS.

### Twenty-four-hour Holter ECG and heart rate variability

During the 24-h Holter ECG evaluation, 8 patients had rare extra ventricular beats, 3 patients had 3 to 5 consecutive VES in a row that was called non-sustained ventricular tachycardia (VT), and 2 patients had rare extra supraventricular beats in the patient group, whereas there were 5 participants with VES and 11 participants with SVE in the control group.

Statistically significant differences were found in especially the SDNN, SDNNI, SDANN, and TINN, which are the time domain components of the HRV in the HUS patients, who had impaired and declined HRV values compared to the control group. The frequency domain HRV parameters were also lower than in the control group, and the difference was significant for the VLF, while *P*-value was > 0.05 for the HF, LF, and LF/HF ratio. Despite the non-significant difference, the LF was much lower (*P* = 0.054) compared to the HF (*P* = 0.124). There was no statistically significant difference between the HRV parameters measured in the patients with and without cardiomyopathy, and with typical and atypical HUS (*P* > 0.05), either. The HRV parameters comprising both the time domain and frequency domain features are summed up in Table 2.

**Table 2:** Heart rate variability parameters including both time domain and frequency domain components and physical examination measurements and comparison between HUS, and control group

**Group**	**HUS **(*N* = 25)	**CONTROL HEALTHY GROUP (*N*** **= 51)**	***P***
	Median (Q1–Q3)	Median (Q1–Q3)	
Heart rate minimum	62,000 [38,000–99,000]	58,000 [44,000–112,000]	0.607
Heart rate maximum	163,000 [135,000–190,000]	172,000 [121,000–188,000]	0.130
Heart rate mean	101,000 [65,000–137,000]	95,000 [65,000–134,000]	0.143
SDNN	93,000 [40,000–299,000]	129,700 [37,800–245,200]	**0.019**
SDNNi	54,500 [21,000–137,000]	66,000 [29,200–196,800]	**0.032**
SDANN	77,000 [36,000–252,000]	105,000 [17,000–230,000]	**0.042**
RMSSD	45,500 [16,800–135,100]	54,200 [18,300–170,800]	0.066
pNN50%	11,000 [0,000–54,000]	18,000 [0,000–43,000]	0.115
VLF	2,134,000 [424,000–9,808,000]	3,157,000 [509,000–8,178,000]	**0.034**
HF	928,000 [58,000–7,613,000]	1,359,000 [184,000–9,026,000]	0.124
LF	1,017,000 [122,000–4,990,000]	1,621,000 [424,000–4,682,000]	0.054
LF/HF	1000 [0.300–2500]	1000 [0.200–4300]	0.996
TINN	421,800 [171,800–1,250,000]	546,800 [125,000–1,078,000]	0.049
Triangular INDEX	27,000 [11,000–80,000]	34,000 [6000–69,000]	0.086

*N* number, *HUS* hemolytic uremic syndrome

The discriminating power of HRV and ECG parameters on patients with HUS was evaluated. The predictivity of the SDNN, SDNNI, SDANN, VLF, and TINN in HRV, and PR, and QTd in ECG was higher according to others. According to this model, cutoff values were calculated. Individuals with values below these HRV and PR interval cutoff parameters and with values above QTd cutoff parameter were classified as HUS. All cutoff and *P* values are explained in Table 3.

**Table 3:** Cutoff parameters of HRV and ECG values for classifying as HUS

	AUC	*P* value	Cutoff values
SDNN	0.666	**0.013**	≤ 122.5
SDNNi	0.652	**0.031**	≤ 55
SDANN	0.644	**0.029**	≤ 101
VLF	0.650	**0.040**	≤ 2798
TINN	0.640	**0.044**	≤ 456
PR (ms)	0.694	**0.003**	≤ 100
QTc	0.639	0.057	> 428
QTd	0.740	** < 0.001**	> 75

*AUC* area under curve, *ECG* electrocardiography, *HUS* hemolytic uremic syndrome, *ms*, milliseconds

### Electrocardiography parameters

When the electrocardiographic findings were evaluated by comparing the patient and control groups, it was observed that there was a significant decrease in the PR distance, while a significant increase in the QTc and QTd values was detected in the patient group (Table 4). Multiple logistic regression analysis approved these findings. This was compatible with the ventricular repolarization impairment in these patients.

**Table 4:** ECG parameters between patients with HUS and the control group

**Group**	**HUS **(*N* = 25)	**CONTROL GROUP **(*N* = 51)	***P***
	Median (Q1–Q3)	Median (Q1–Q3)	
**PR ms**	118,000 [94,000–125,500]	125,000 [118,000–140,000]	**0.003**
**QRSms**	73,000 [48,000– 90,500]	85,000 [78,000–90,000]	0.078
**QT**	340,000 [290,500–363,000]	330,000 [310,000–350,000]	0.929
**QTc**	430,000 [410, 000–440, 000]	411,000 [400, 000–428, 000]	**0.043**
**QTd**	89,000 [66,000–104,000]	56,000 [32, 000–78, 000]	**0.001**
**Tp_e**	60,000 [38,000–82,000]	52,000 [41,000–68,000]	0.081
**Tp-e dispersion**	42,000 [25,000–51,000]	32,000 [17,000–44,000]	0.110
**Tp-e/QT ratio**	0.180 [0,115–0,240]	0.150 [0.120–0.200]	0.387

*N* number, *ms* milliseconds, *HUS* hemolytic uremic syndrome

### Laboratory biomarkers and correlations

In the HUS group, there was a negative correlation between the LF/HF ratio and urea, creatinine, sodium, and potassium values (*r* = −0.761, *P* = 0.000; *r* = −0.578, *P* = 0.005; *r* = −0.439, *P* = 0.041; and *r* = −0.513, *P* = 0.015, respectively). A negative correlation was also present between the LF/HF ratio and LDH (*r* = −0.491, *P* = 0.024).

There was no significant correlation between the blood count parameters, thyroid hormones, CRP, haptoglobin, C3, C4, coagulation values, or cardiac biomarkers (CK-MB and troponin I) in the HUS and HRV parameters (*P* > 0.05).

### Antiarrhythmic treatment

Patients with non-sustained VT attacks with/without QTc prolongation were treated with a beta-blocker (metoprolol). All patients were followed up regularly in the cardiology clinic after discharged. Two patients with prominent cardiomyopathy and associated ventricular arrhythmia died after 12 months from diagnosis. The cause was systematic impairment rather than mortal tachycardia attacks. No patient died because of arrhythmia till now after the acute period.

## Discussion

In this study, the decrease in HRV parameters was significant in the HUS patients compared to those in the control group. An impairment in the HRV can be observed as renal failure parameters, sodium, and potassium values worsen. When the ECG and blood pressure parameters were analyzed, it was seen that the PR ms, QTd, and systolic blood pressure were the variables that showed the most significant differences between the patient and control groups. While the PR decreased in the patient group, the QTd and systolic blood pressure increased significantly. When all of these are evaluated together, it is seen that care should be taken in terms of autonomic nervous system dysfunction, QTd prolongation and arrhythmia, and cardiovascular and other systemic effects in patients diagnosed with HUS. After reviewing the literature, it appears this is the first study assessing these values in HUS.

HUS is a clinically diagnosed systemic disease with acute renal failure and deterioration in hematological parameters. Cardiovascular involvement may present as microvascular injury, and subsequent microvascular injury–related diseases like occlusion-related cardiomyopathies and/or myocardial infarction could occur in these patients [3]. Since it is a systemic disease, it can also affect the nervous system as a result of microvascular occlusion and ischemia in all vessels, resulting in autonomic nervous system dysfunction, autonomic neuropathy, and activation of the sympathetic system [6, 14]. Dysfunction of the autonomic nervous system could lead to abnormalities in the sympathovagal balance. This interaction might cause respiratory, cardiovascular, gastrointestinal, genitourinary, and neurologic system problems [15].

A non-invasive method of analyzing autonomic function is the usage of the HRV, which is related to modifications in the relaxation and stress status of individuals within the sympathetic and parasympathetic autonomic nervous system. Normal ranges for the HRV in children can be provided from adult and healthy children studies. Prognostically, reductions in the HRV are independent predictors of overall mortality, mortality from heart failure, sudden cardiac death, ventricular arrhythmias, and the need for transplant [14].

The time domain and frequency domain features of the HRV like the SDNN, SDNNI, SDANN, TINN, and VLF, which indicate parasympathetic activity, decreased significantly in the patients with HUS. The LF shows both sympathetic and parasympathetic activity. The LF/HF ratio may guess the ratio between the sympathetic nervous system and parasympathetic nervous system, but the median value of the LF/HF ratio was equal between the groups. This could have been due to a simultaneous LF and HF value decrease in each group. An increase in sympathetic activity could have also been another expected cause for this. Although the triangular index was not significantly different between the two groups, it was also diminished in the HUS group. All of these parameters could lead to multisystemic dysfunction including cardiac, neurologic, and gastrointestinal systems that are in contact with the autonomic nervous system.

Arrhythmias and related effects are less common in HUS [4]. In some case reports, ventricular tachycardia and, although rare, sudden cardiac death have been reported [16]. Predicting which of the patients diagnosed with HUS may be at risk of cardiac arrhythmia will guide physicians about treatment management during the follow-up. The excess of cardiac involvement in these patients may also be related to dysfunction of the autonomic nervous system. Along with close follow-up, 24-h Holter ECG and antiarrhythmic drugs should be considered in patients with increased cardiovascular risk. At the same time, these patients may be predisposed in terms of neurological symptoms and central nervous system involvement [17, 18]. Especially considering that peripheral and autonomic nervous system involvement may be subclinical, the HRV seems very useful as a non-invasive parameter that can be used to evaluate patients in this respect. If these patients are caught in the subclinical period, additional measures can be taken to prevent progression [17].

Looking at the ECG findings, it can be said that QT prolongation is especially in the risk group in terms of arrhythmia and Torsades de Pointes, considering that the QTc, QTd, and PR distances changed in the patient group. This shows that ECG evaluation is important in patients [19].

Considering the laboratory data and correlations, the HRV values decreased as the renal failure parameters increased. In a study conducted in patients with chronic renal failure, a decreased HRV was found to be compatible with increased mortality risk, and this supports the data in the HUS study [20].

To our knowledge, there are no studies about measurement of the HRV in patients with HUS. Further large sample studies should be conducted to evaluate the clinical use of the HRV in these clinical conditions and also its associated neuroautonomic effects.

### Limitations

This was a single-center study and these diseases are very rare. Other restrictions rise from the approach of 24-h Holter ECG, which can be changed by all movement and the attitude of participants and sleep conditions.

In conclusion, patients with HUS may have autonomic nervous system dysfunction. HRV measurement is a non-invasive method that can evaluate this. It can be thought that there may be an increased risk of cardiovascular events and arrhythmias due to the deterioration of the balance between the sympathetic and parasympathetic systems in patients with HUS. Other systems may also be affected, including neurological events. Thus, 24-h Holter ECG with 12 lead electrocardiography should be considered in these patients. In patients diagnosed with HUS, it may be better to take an ECG and screen patients for cardiac events, and monitoring them more closely should be considered. Larger studies with larger numbers of patients may be reasonable to determine a cutoff value of the HRV parameters.

## Data Availability

The data that support the findings of this study are not openly available due to reasons of sensitivity and are available from the corresponding author upon reasonable request.

## References

[1] Cody EM, Dixon BP (2019). Hemolytic uremic syndrome. Pediatr Clin North Am.

[2] Razzaq S (2006). Hemolytic uremic syndrome: an emerging health risk. Am Fam Physician.

[3] Noris M, Remuzzi G (2014). Cardiovascular complications in atypical haemolytic uraemic syndrome. Nat Rev Nephrol.

[4] Sanders E (2021). Cardiac manifestation among children with hemolytic uremic syndrome. J Pediatr.

[5] Malik M, Camm AJ (1993). Components of heart rate variability–what they really mean and what we really measure. Am J Cardiol.

[6] Cygankiewicz I, Zareba W (2013). Heart rate variability. Handb Clin Neurol.

[7] Kasamaki Y (2011). Automated versus manual measurement of the QT interval and corrected QT interval. Ann Noninvasive Electrocardiol.

[8] Ciobanu A (2014). Dispersion of ventricular repolarization in relation to cardiovascular risk factors in hypertension. J Med Life.

[9] Electrophysiology TF (1996). Heart rate variability: standards of measurement, physiological interpretation and clinical use. Task Force of the European Society of Cardiology and the North American Society of Pacing and Electrophysiology. Circulation.

[10] Asarcikli LD (2022). Heart rate variability and cardiac autonomic functions in post-COVID period. J Interv Card Electrophysiol.

[11] Berntson GG (1997). Heart rate variability: origins, methods, and interpretive caveats. Psychophysiology.

[12] Sassi R (2015). Advances in heart rate variability signal analysis: joint position statement by the e-Cardiology ESC Working Group and the European Heart Rhythm Association co-endorsed by the Asia Pacific Heart Rhythm Society. Europace.

[13] Nunan D, Sandercock GR, Brodie DA (2010). A quantitative systematic review of normal values for short-term heart rate variability in healthy adults. Pacing Clin Electrophysiol.

[14] Fauchier L (1999). Prognostic value of heart rate variability for sudden death and major arrhythmic events in patients with idiopathic dilated cardiomyopathy. J Am Coll Cardiol.

[15] Stein PK (2003). Heart rate turbulence: explorations of an emerging risk factor. J Cardiovasc Electrophysiol.

[16] Yesilbas O (2020). Sudden cardiac arrest and malignant ventricular tachycardia in an 8-year-old pediatric patient who has hemolytic uremic syndrome associated with Shiga toxin-producing Escherichia coli. J Pediatr Intensive Care.

[17] Goldstein J, Nuñez-Goluboay K, Pinto A (2021). Therapeutic strategies to protect the central nervous system against Shiga toxin from enterohemorrhagic Escherichia coli. Curr Neuropharmacol.

[18] Mansour MA (2023). Hemolytic uremic syndrome with central nervous system manifestations, a case report and literature review. Radiol Case Rep.

[19] Filip C (2020). Cardiovascular complications of hemolytic uremic syndrome in children. Maedica (Bucur).

[20] Thio CHL (2018). Heart rate variability and its relation to chronic kidney disease: results from the PREVEND Study. Psychosom Med.

